# Neuropeptide natalisin regulates reproductive behaviors in *Spodoptera frugiperda*

**DOI:** 10.1038/s41598-024-66031-y

**Published:** 2024-07-02

**Authors:** Wei Gong, Jun-Hong Linghu, Hui-Min Xu, Li-Lin Luo, Guy Smagghe, Tong-Xian Liu, Shun-Hua Gui

**Affiliations:** 1 Guizhou Provincial Key Laboratory for Agricultural Pest Management of the Mountainous Region, Institute of Entomology, Guizhou University, Guiyang, 550025 Guizhou China; 2 Guizhou Institute of Biology, Guizhou Academy of Sciences, Guiyang, China; 3 Institute of Plant Health and Medicine, Guizhou University, Guiyang, China

**Keywords:** *Spodoptera frugiperda*, Fall armyworm, Neuropeptide, Natalisin, Reproduction, Sexual activity, Mating behavior, Calling, Courtship, Biochemistry, Physiology

## Abstract

Natalisin (NTL) is a conserved neuropeptide, only present in insects, that has been reported to regulate their sexual activity. In this study, we investigated the involvement of NTL in the reproductive behaviors of a major invasive pest, *Spodoptera frugiperda*. We identified NTL precursor-encoded transcripts, and evaluated their transcript levels in different stages and tissues of *S. frugiperda*. The results showed that the *NTL* transcript level was expressed in both male and female pupae and both male and female adults in the later stage. It was highly expressed in male pupae, 3-day-old male and female adults, and 5-day-old male adults. In different tissues, the expression level is higher in the male and female adult brain and male testis. Immunohistochemical staining of the brain of *S. frugiperda* female and male adults revealed that three pairs of brain neurons of *S. frugiperda* adults of both sexes secreted and expressed NTL. To study the role of NTL in reproductive behaviors, *NTL* was silenced in *S. frugiperda* male and female adults by RNA interference (RNAi) technology, the results showed that silencing NTL could significantly affect the sexual activity behavior of the adults, reducing the calling rate of females, the courtship rate of males, and the mating rate. In summary, this study emphasizes the important role of NTL in regulating the mating behavior and sexual activity of *S. frugiperda* in both male and female adults, potentially laying a foundation to employ NTL as a new insect-specific target to control populations of pest insects.

*Spodoptera frugiperda* (J. E. Smith) (Lepidoptera: Noctuidae), also known as the fall armyworm, is one of the world’s major invasive agricultural pests. It is native to tropical and subtropical regions of North and South America^1^, but it is now distributed in dozens of countries around the globe due to its strong migratory and reproductive abilities^2–5^. For instance, the females have large egg production: the number of eggs per mass varies around 100 to 200, and the total egg production per female averages about 1500 with a maximum of over 2000^6^. In China, *S. frugiperda* migrates into the Yangtze River Delta with an area of about 350,000 square kilometers^7^, and Wang et al.^8^ reported that *S. frugiperda* may invade countries in southern Europe from North African countries through the wind as a medium whereby the adults can migrate hundreds of kilometers in one night and fly more than 500 km in one generation. This armyworm poses a serious threat to global agriculture due to its high reproductive capacity^9^. Therefore, it is urgent to develop specific control agents for *S. frugiperda*^10^.

Insect neuropeptides have long been considered ideal targets for control agents^11–14^. The neuropeptide natalisin (NTL) is part of the insects’ peptidergic system, which is the genetic network that uses small peptides as neurotransmitters to chemically relay messages throughout the body. Interestingly, NTL is unique to insects and arthropods and has evolved with them^15–17^. NTL appears to be related to a neuropeptide called tachykinin that is in mammals and invertebrates. While tachykinin is involved with various biological processes, including the control of blood flow in mammals, NTL is linked to reproductive function and mating behavior in insects and arthropods. For instance, in the insects, *Drosophila melanogaster* and *Tribolium castaneum*, NTL has been reported to be involved in the regulation of reproductive behaviors^15^. Studies in the oriental fruit fly, *Bactrocera dorsalis* (Hendel), have likewise confirmed that NTL has a similar function. Subsequent silencing of the NTL or its receptor in *B. dorsalis* using RNAi significantly reduced the mating rate regardless of whether females or males were treated, but egg production in successfully mated females was not significantly affected^10,18^. Recently, researchers successfully silenced the *S. litura* NTL using bacterial-mediated RNAi technology and found that the courtship and mating behaviors of the *S. litura* were significantly inhibited, and egg-laying was significantly reduced^19^. Although NTL has been shown to be involved in regulating reproduction in these species, whether NTL also regulates the related functions of *S. frugiperda* remains to be verified.

In this study, we studied the molecular characteristics of NTL in *S. frugiperda*. We analyzed the expression of *NTL* in different developmental stages and tissues of *S. frugiperda* by quantitative real-time PCR (qRT-PCR), and analyzed the neuronal localization in the brain of adults *S. frugiperda* by immunohistochemistry. In addition, this study used a nanomaterial-mediated dsRNA delivery system to silence *NTL*, revealing the function of the NTL signaling system. In the future, since NTL is unique in insects and an important regulator of the sexual activity in insects, our data may inspire the use of the neuropeptide NTL as a new insect-specific target to control populations of *S. frugiperda* and other pest insects.

## Methods

### Insects

Briefly, the *S. frugiperda* was reared under standard rearing conditions of (25 ± 1) °C, (65 ± 5)% relative humidity, and a photoperiod with 14 h light and 10 h darkness in the laboratory. Adults were reared in 40 cm × 60 cm × 40 cm insect rearing cages. The eggs were artificially collected and transferred to a plastic incubator (10 cm × 5 cm × 6 cm) until the larvae hatched. The *S. frugiperda* larvae were reared individually in plastic cups (3 cm in height and 2 cm in diameter), and fresh artificial feed^20^ was changed daily until pupation. The adults were given a 10% (V: V) honey solution. In our population, the pupal stage of *S. frugiperda* is about 12 days, and the adult usually enters the peak of mating on the third day after emergence.

### NTL sequence analysis in *S. frugiperda*

The sequence information of *S. frugiperda* NTL (SfNTL) was derived from our transcriptome data (unpublished), and then aligned with the NTL precursor sequences from other species by using the Clustal X2 software^21^, with default settings. The signal peptide of NTL precursor was predicted using the SignalP server (https://services.healthtech.dtu.dk/service.php?SignalP-5.0), and sequence logos for the C-terminal motifs of NTL were generated by WebLogo^22^.

### Gene expression in different developmental stages and tissues

In order to study the specific expression of *SfNTL* in different developmental stages and tissues at the mRNA level, we collected 10-day-old male and female pupae, and 1-,3-, 5-day-old male and female adults. At the same time, the brain, gut, fat body, testis and ovary of 5-day-old male and female adults were dissected in chilled phosphate buffered saline (PBS, pH 7.4). In different developmental stages, each sample consisted of 3 individuals and was repeated 3–5 times. Fifteen samples from each tissue were used as a biological replicate, with a total of 3–5 biological replicates. Total RNA was extracted using Trizol (Sangon Biotech, China). The quality and concentration of RNA were detected by 1% agarose gel electrophoresis and Nanodrop 2000 spectrophotometer (Thermo Fisher Scientific, USA). Then 1 μg RNA was reversely transcribed into cDNA according to the kit instructions (Genstar, China).

Analysis was performed with CFX 96™ Real-Time system and the CFX manager software (Bio-Rad, USA) in a 10 μL reaction system, containing 0.5 μL of cDNA samples (approximately 1 μg/μL), 5 μL of 2X RealStar SYBR qPCR Mix (Genstar, Beijing, China), 0.3 μL of each primer (10 μM), and 3.9 μL of nuclease-free water. The thermal cycling program included an initial denaturation at 95 °C for 2 min, followed by 40 cycles at 95 °C for 15 s and 60 °C for 30 s. To ensure the specificity and consistency of all generated products, all reactions were analyzed by melting curves at 60–95 °C. The primers used in qRT-PCR (Table S1) were validated by standard curves based on the dilution of the cDNA sequences to determine the amplification efficiency of the primers, and used glyceraldehyde-3-phosphate dehydrogenase (*GAPDH*) as internal reference gene for different developmental stages^23^, *RPL10* as an internal reference gene for different tissues, because they have excellent stability in the *S. frugiperda*^24^. Relative gene expression levels were calculated from the expression levels of the reference genes by CFX manager software.

### Antibodies and immunohistochemistry

Due to the high structural conservation of *S. frugiperda* NTL mature peptide and *D. melanogaster* NTL mature peptide DmNTL4 (HRNLFQVDDPFFATRamide), rabbit anti-DmNTL4 polyclonal antibody (anti-DmNTL4) was used for immunohistochemical analysis, which was donated by Professor Jiang Hongbo of Southwest University^15^. The central nervous system (CNS) of 5-day-old male and female adults were dissected in chilled PBS, fixed with fresh 4% paraformaldehyde for 4 °C or overnight, and rinsed with 1% PBST (PBS + Triton X-100) 6 times (20 min once). Tissues were placed in a blocking solution (10% normal goat serum (NGS), NGS diluted in 1% PBST) at 4 °C overnight, while primary antibodies were added, and the tissues were incubated with the primary antibody (anti-DmNTL4; 1:500) for 5 days at 4 °C with gentle shaking, and then washed six times with 1% PBST for 20 min each. We used 1% PBST instead of primary antibody as a negative control. Subsequently, the tissues were incubated with goat anti-rabbit immunoglobulin G antibody labeled with Dylight 488 (1:1000, 1%PBST); for 3 days at 4 °C and the samples were washed six times with 1% PBST for 20 min each. Tissues were dehydrated sequentially using a series of ethanol, and afterwards dehydrated brain tissues were placed in methyl salicylate with overnight clearing at 4 °C, and then mounted on clean slides using neutral gum. Image microscopy was acquired with the Nikon A1R Confocal Microscope System (Nikon Instruments, USA).

### RNAi bioassay

Based on the SfNTL precursor sequence, primers were designed to amplify specific regions and the T7 promoter was added before the primers to synthesize dsRNA targeting *SfNTL*. The PCR products were purified and verified by DNA sequencing. The dsRNA was then synthesized using the Transcript Aid T7 High Yield Transcription Kit (Thermo Scientific) according to the manufacturer's instructions. Confirmation of the integrity of the dsRNA product by agarose gel electrophoresis (1%). The dsRNA of green fluorescent protein (GFP) was used as control.

We used a nanocarrier (star polycation, SPc)-mediated dsRNA delivery system for RNAi of *S. frugiperda* adults^25^. The 4 µg of dsRNA (dsRNA-NTL or dsRNA-GFP) was mixed with nanomaterials in a 1:1 mass ratio. The mixture was injected the intersegmental membrane in the 8th-9th abdomen of 2-day-old adults using microinjection. Then, at 24 h after injection of dsRNA, samples were collected for detecting the RNAi effect of the target gene. The dsRNA-GFP was used as control. *GAPDH* was selected as the reference gene. Each treatment contains three independent samples. The RNAi efficiency was calculated by qRT-PCR, and the method was the same as above.

### Behavioral assays

All behavioral observations were performed under standard artificial climate conditions. After eclosion, the adult was reared individually in a plastic box. dsRNA-NTL or dsRNA-GFP was injected 2 days after eclosion, and single-head pairing was performed 30 min before the dark period on the third day to observe reproductive behavior. The experiment consisted of four treatments:

1. dsRNA-GFP treated female crossed with wild virgin male (30 pairs)
2. dsRNA-NTL treated female crossed with wild virgin male (30 pairs)
3. dsRNA-NTL treated male crossed with wild virgin female (30 pairs)
4. dsRNA-GFP treated male crossed with wild virgin female (30 pairs)

The treated insects were placed in a transparent container with a height of 4 cm and a diameter of 5.6 cm for single-head pairing. After the above treatment, all paired insects were quickly and rapidly scanned every 10 min and the following behaviors were recorded: male courtship, female calling and mating. The description of reproductive behavior was recorded according to Wang’s description^19^. Immediately after the end of a dark period of mating, the male adult was removed and the number of eggs laid in the next 3 days was recorded. Statistical analyses of mating rate, calling rate and courtship rate were carried out using Fisher’s exact test, and independent samples t-tests were used to analyze the significance of differences in calling latency, courtship latency, mating latency, egg production and mating duration^10^.

### Effects of male* S. frugiperda* on calling behavior of female insects

The 3-day-old virgin females and males, and the 3-day-old virgin females were placed in transparent containers with a height of 4 cm and a diameter of 5.6 cm for observation. After the above treatment, all paired insects were scanned rapidly every 10 min and the calling behavior of female insects was recorded.

### Statistical analysis

The SPSS version 22 software was used for all analyses. Shapiro–Wilk test was first used to test for normality in the data sets. When normally distributed (Shapiro–Wilk tests: *P* > 0.05), an independent Student’s t test was used to compare the test vs the control. When the data was not distributed normally (Shapiro–Wilk tests: *P* ≤ 0.05), a nonparametric Mann–Whitney-U test for different groups was used for comparison. In brief, calling latency, courtship latency, mating latency, mating duration, egg production from different treatment groups were compared using the Mann–Whitney-U test or Student’s t test. All reproductive behavior rate use the analysis method of Fisher's exact test.

## Results

### Sequence analysis and molecular characterization of NTL

**Figure 1:**
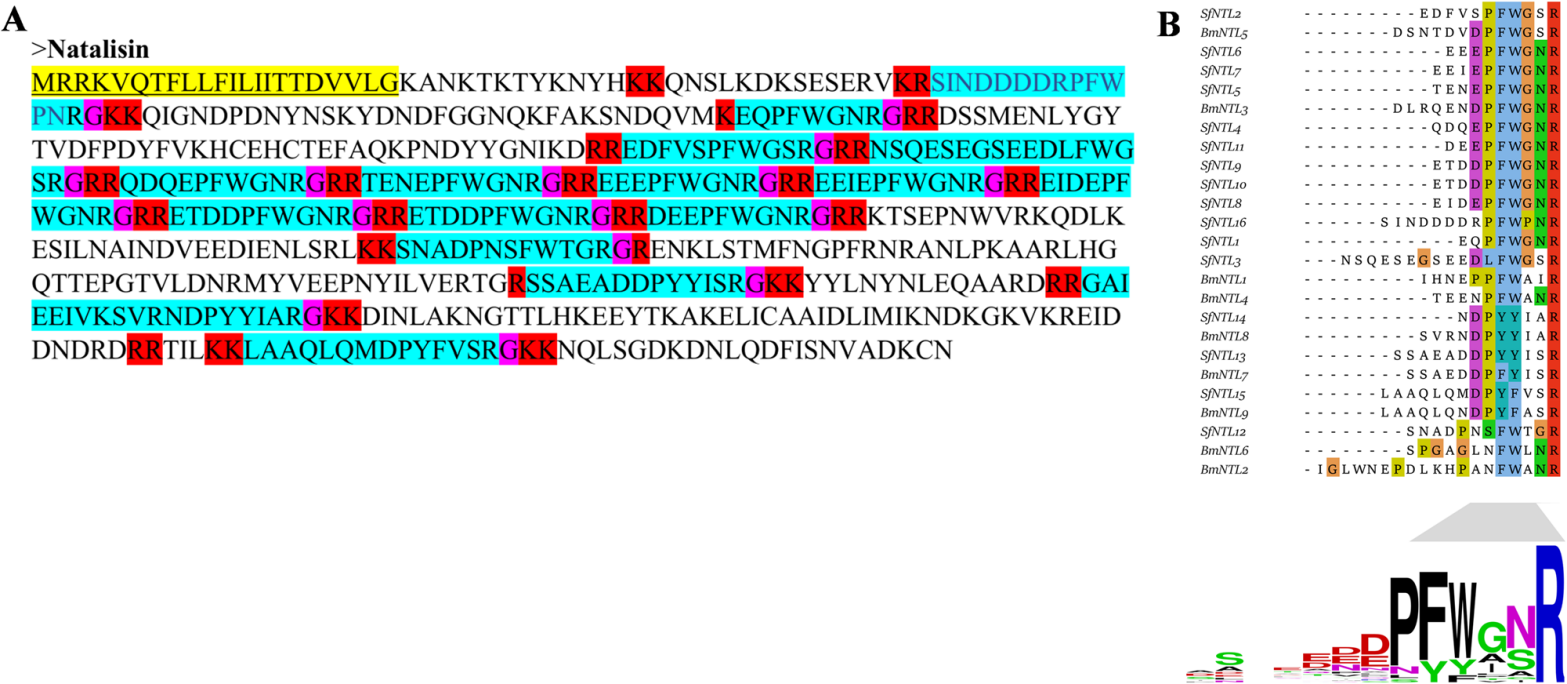
Deduced amino acid sequence of the natalisin (NTL) precursor and prediction of the mature peptide. (**A**) Deduced amino acid sequence of *Spodoptera frugiperda* NTL. The coding sequences are the signal peptide (with yellow sequence and underlined sequence) and the predicted NTL peptide (blue sequence). Predicted amidation signal (purple sequence) with a dibasic cleavage site (red sequence) is predicted. (**B**) Comparison of consensus sequences of NTL putative mature peptides of *S. frugiperda* (Sf) and *Bombyx mori* (Bm). The calculated consensus logo is shown at the bottom.

Based on RNA-seq technology, the full-length mRNA sequence of NTL gene was obtained from the transcriptome of *S. frugiperda*. This mRNA contained a putative open reading frame (ORF) of 1629 nucleotides encoding a 543-amino acid protein (Fig. 1A). The first 22 underlined amino acid residues of the precursor were predicted to be secreted signal peptides. Sixteen putative *S. frugiperda* NTL peptides were identified by flanking dibasic cleavage sites (a combination of K and R). In addition, an amidation C-terminus of each mature peptide was determined by a typical amidation site (G). Display consensus sequence DDPFxPxRamide (‘x’ represents the mutation site, ‘amide’ represents the amidated C-terminus) of the *S. frugiperda* and *Bombyx mori* NTL mature peptides was presented in Fig. 1B. The C-terminus of the 13 putative mature peptides contained the conserved motif FxxxRamide of the insect NTL, and three of the putative neuropeptides contained the lepidopteran-specific NTL conserved motif YxxxRamide.

### The expression of NTL in *S. frugiperda* at different stages and tissues

**Figure 2:**
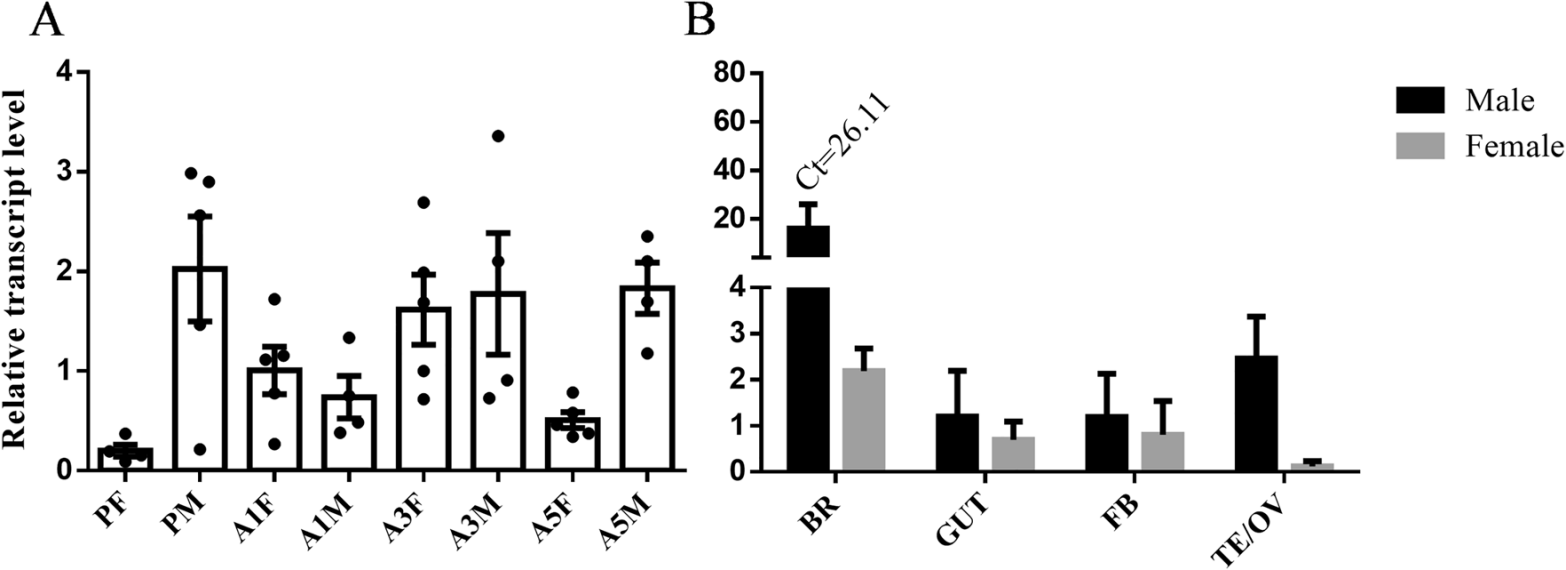
The relative expression of *SfNTL* in different developmental stages and different tissues of *Spodoptera frugiperda.* The data of different developmental stages were standardized with *GAPDH* as the internal reference, and the data of different tissues were standardized with *RPL10* as the internal reference, expressed as fold-changes compared with calibrator ± SE (n = 3–5). PF (10-day-old female pupae); PM (10-day-old male pupae); A1F, A3F, A5F (1-, 3-, 5-day-old virgin female adults); A1M, A3M, A5M (1-, 3-, 5-day-old virgin male adults) Abbreviations used on x-axis: *BR* brain, *FB* fat body, *GUT* gut, *OV* ovary, *TE* testis.

In order to study the expression dynamics of *SfNTL* genes in the *S. frugiperda* at different stages and tissues of adults or pupae, we analyzed the transcript levels of *NTL* using qRT-PCR. The results are shown in Fig. 2A. *SfNTL* was expressed in all test stages of adults and pupae, with higher expression in the late stage of male pupae, 3-day-old male and female adults, and 5-day-old male adults. Subsequently, we examined the expression of *SfNTL* in different tissues and found that *SfNTL* was highly expressed in the brain of male and female adults and male testis (Fig. 2B).

### Localization of NTL in the CNS of the male and female adult

**Figure 3:**
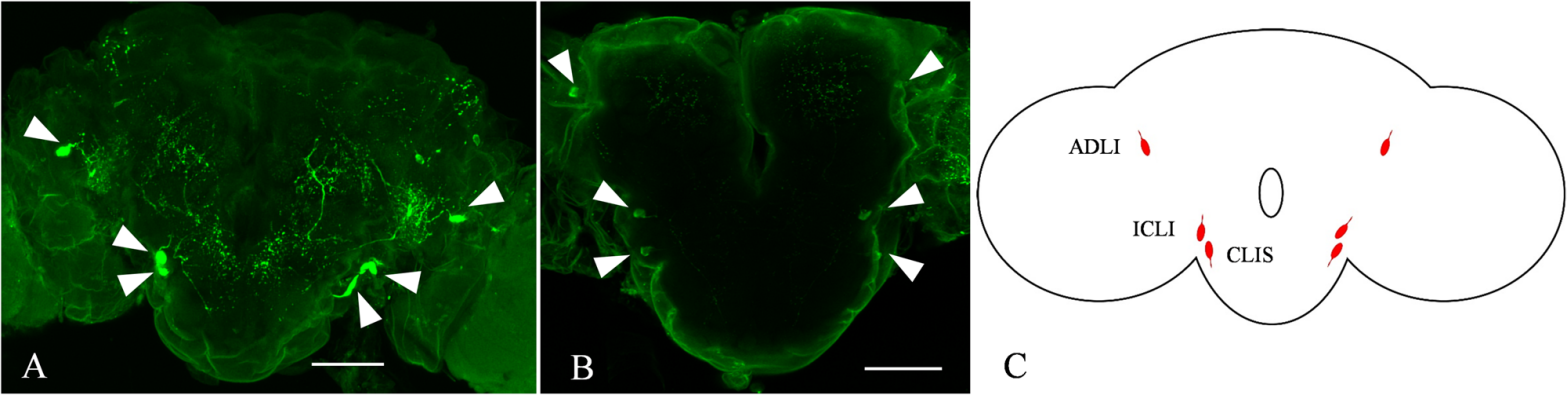
The distribution of SfNTL in the brain of *Spodoptera frugiperda* 5-day-old male and female adults. (**A**) Anti-DmNTL4-labeled nerves in the brain of *S. frugiperda* adults: male (**B**) and female. (**C**) The distribution pattern of NTL in the brain of *S. frugiperda* adults: male and female. The white triangle indicated the neurons expressing NTL. The scale is 100 μm.

In order to study the localization of NTL-expressing neurons in the CNS of *S. frugiperda* male and female adults, we conducted a whole-mount immunohistochemical incubation. In the male (Fig. 3A) and female brains (Fig. 3B), the results were equal and showed that the antibody detected three pairs of significantly expressed neurons, including a pair of anterior dorsolateral interneurons (ADLI) and a pair of inferior contralateral interneurons (ICLI) and detected in a pair of neural processes innervating the suboesophageal ganglion (SOG) and referred to as the contralateral interneuron (CLIS) of the SOG (Fig. 3C). Moreover, we also found neuronal projections in the thoracic and abdominal ganglia of the ventral nerve cord (Fig. S1).

### Effect of SfNTL knockdown on mating behavior with female calling and male courtship rates, and fecundity

**Figure 4:**
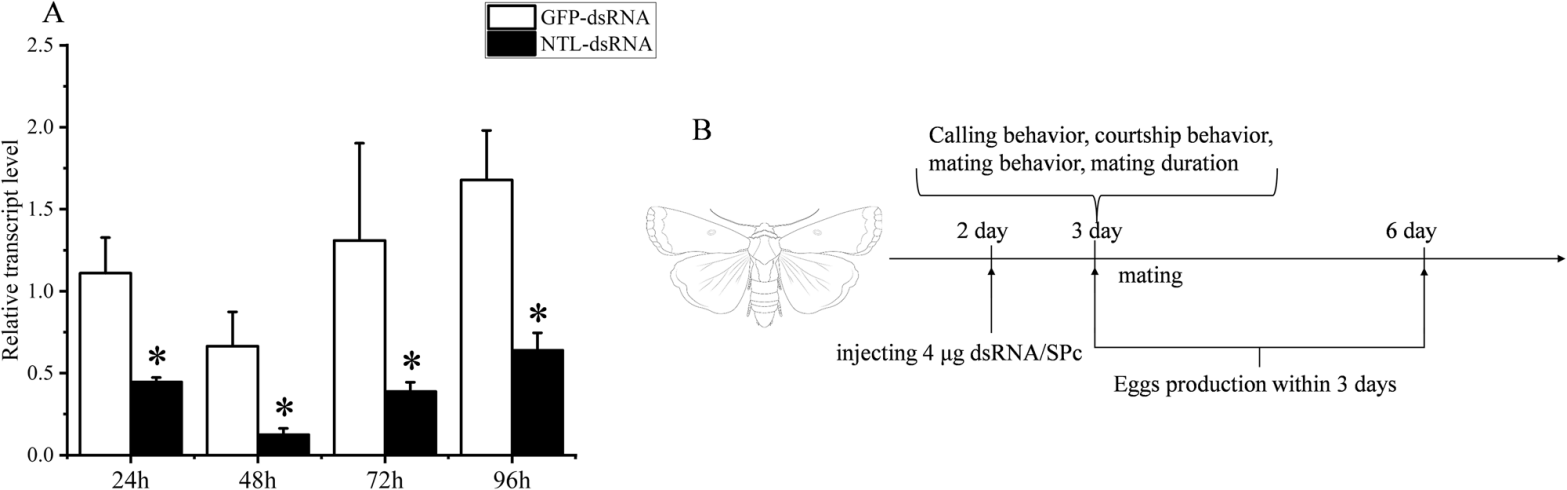
Detection of *SfNTL* silencing efficiency mediated by dsRNA of *Spodoptera frugiperda* and observation flow chart of interfering with neuropeptide NTL behavior of *S. frugiperda* male and female adults. (**A**) The effect of natalisin-double-strand DNA (dsRNA-NTL) injection on NTL precursor transcripts in *S. frugiperda* adults. The knockdown efficiency was measured after injection of dsRNA/SPc. Date are means ± SE, n = 3–5. The asterisk indicated that the relative expression level (*P < 0.05) was significantly different. Abbreviation: GFP, green fluorescent protein. (**B**) On the second day of eclosion, dsRNA/SPc was injected, and the unmated adults injected with dsRNA were mated with the wild type on the third day. The calling, courtship, and mating behaviors were recorded every 10 min. After a dark period, the male was taken out and the number of eggs laid by the female in three days was recorded.

**Figure 5:**
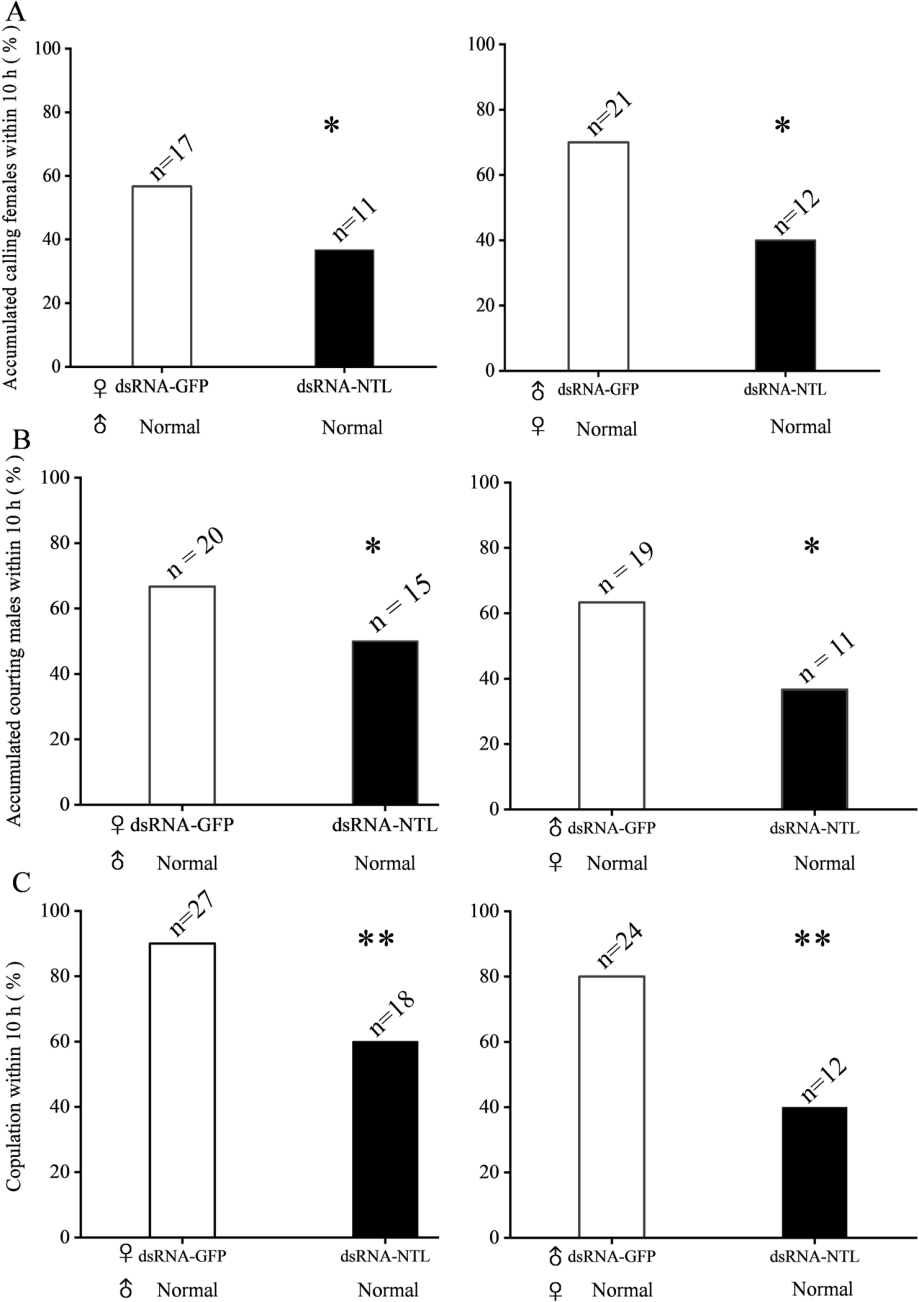
Effects of natalisin double-stranded DNA (dsRNA-NTL) injection on the reproductive behavior rate of *Spodoptera frugiperd* male and female adults. The data were analyzed by Fisher 's exact test. *P < 0.05, **P < 0.01. (**A**) Calling behavior rate of dsRNA-NTL adults and untreated virgin adults. (**B**) Rate of courtship behavior between dsRNA-NTL adults and untreated virgin adults (**C**) Rate of single mating between dsRNA-NTL adults and untreated virgin adults. Abbreviation: GFP, green fluorescent protein.

**Figure 6:**
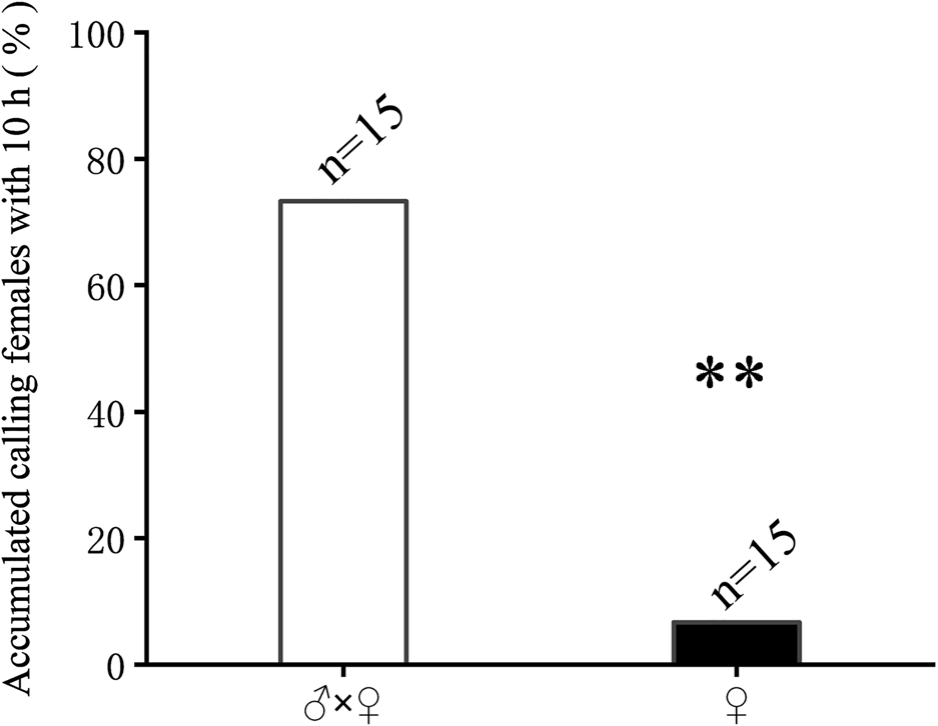
Effects of male *Spodoptera frugiperd* on calling behavior of female insects. Effect of presence or absence of male insects on female calling rate. The data were analyzed by Fisher’s exact test. *P < 0.05, **P < 0.01. n = 15.

We used RNAi to study the effect of NTL precursor on the reproductive behavior of the male and female adults of *S. frugiperda.* The results showed that the mRNA levels of NTL were significantly decreased by 60%, 81%, 70% and 62% at 24 h, 48 h, 72 h and 96 h after NTL knockout, respectively (Fig. 4A). Subsequently, we followed the calling behavior in the female, courtship behavior in the male, mating behavior, mating duration and fecundity (Fig. 4B). It was clear that as compared with the *S. frugiperda* treated with dsRNA-GFP, the rates of calling behavior, courtship behavior and mating behavior in *S. frugiperda* treated with dsRNA-NTL were less in the 10 h dark photoperiod (P < 0.05, Fisher’s exact test) (Fig. 5). The calling behavior rate of dsRNA-NTL treated females (37%) was significantly lower than that of dsRNA-GFP treated females (57%, P < 0.05, Fisher’s exact test), the calling behavior rate of females in the dsRNA-NTL treatment males (40%) was significantly lower than that in the dsRNA-GFP treatment males (70%, P < 0.05, Fisher’s exact test) (Fig. 5A). The courtship behavior rate of dsRNA-NTL treated females (50%) was significantly lower than that of dsRNA-GFP treated females (67%, P < 0.05, Fisher’s exact test), the rate of courtship behavior in dsRNA-NTL-treated males (37%) was significantly lower than that in dsRNA-GFP treated males (63%, P < 0.05, Fisher’s exact test) (Fig. 5B). The mating rate of dsRNA-NTL treated females (60%) was significantly lower than that of dsRNA-GFP treated females (90%, P < 0.01, Fisher’s exact test), the rate of mate behavior in dsRNA-NTL-treated males (40%) was significantly lower than that in dsRNA-GFP treated males (80%, P < 0.01, Fisher’s exact test) (Fig. 5B). However, there were no differences in the calling latency, courtship latency, mating latency, the number of eggs laid in the first 3 days and the mating duration between the normal mating dsRNA-NTL and dsRNA-GFP *S. frugiperda* (Figs. S2, S3). In order to explore the effect of male *S. frugiperda* on female calling behavior. We set up an experimental scheme for the effect of male *S. frugiperda* on the calling behavior of females. The calling behavior rate of females alone was 7%, and the calling behavior rate of females with males was 73%. The results showed that males could significantly affect the calling behavior of females (P < 0.01) (Fig. 6).

## Discussion

Neuropeptides affect the entire reproductive process of insects from attraction, mating behavior, mating duration, mating rhythm to oviposition^26^. In this study, we focused on the effects of the neuropeptide NTL on the reproductive behavior in *S. frugiperda*. We characterized NTL and found similar precursor structure with other Lepidoptera insects^15^. In most arthropods, NTL precursor has a general motif sequence FxxxR at the C-terminus^15^, However, the motif sequence YxxxR was only found in Lepidoptera insects^12,19^. The further differentiation of NTL may reflect the evolutionary process based on ligand-receptor activity. The NTL peptides of *S. frugiperda* were highly conserved compared with the five NTL peptides of *B. mori* (Fig. 1B). *B. mori* has two NTL receptors, BmNTLR A32 and A33. BmNTLR A32 can be specifically activated by the C-terminal FxxxRamide consensus sequence BmNTL1, BmNTL3, BmNTL4 and BmNTL5, while for BmNTLR A33 this was by BmNTL10 and BmNTL11 with the YxxxRamide consensus sequence^15^. We suspect that the ligand-receptor coevolution of Lepidoptera may be involved in different functional differentiations, and the mature peptide activating receptors of different motifs may affect different physiological functions. Further research should investigate other species in the phylum of arthropoda and the evolutionary diversity between NTL and tachykinin in the animal kingdom.

In order to better understand the function of NTL and in which life stage of the insect it can have a function, we measured the gene expression levels of *SfNTL* in different developmental stages and tissues. Typically, *NTL* was expressed in both male and female pupae and both male and female adults in the later stage (Fig. 2), which agrees with the pattern in other insects^10,19^. In addition, this concurred with the immunostaining results in the male and female brain (Fig. 3A,B), which was similar with previous studies, Jiang et al^15^ also reported that 3–4 pairs of brain neurons secrete NTL in these three model insects. In *B. dorsalis*, 13 pairs of neurons were detected, including a pair of ADLI and ICLI, but no CLIS. In *D. melanogaster*, ADLI and/or ICLI may be associated with mating behavior^15^, as reviewed Schoofs et al.^26^ Therefore, we speculate that NTL may play an important regulatory role in the mating behavior of *S.frugiperda*, and this in both sexes. Further studies are required to clarify the mechanism(s), influencing mating behaviors in the male and female adults.

The mating behavior is a very important process for population reproduction. In our study, NTL affected the reproductive behavior of *S. frugiperda*, where gene-silencing of *NTL* in either the male or female adult significantly reduced the mating rate (Fig. 5), which phenotype is consistent with the results in other insects^10,15,19^. In detail, we examined the calling behavior of females and the courtship behavior of males, and found that after the silencing of *NTL*, the calling rate in the female and the courtship rate in the male were significantly reduced (Fig. 5). Our experiments thus demonstrated that NTL regulates the reproductive behavior of both male and female adults, and this in turn decreased the mating rate. After silencing the male, the calling behavior of the female also decreased significantly. This agrees with the reports in Asian corn borer that the ultrasound produced by males acts as a courtship song^27^, and in dogbane tiger moth that males without pheromones and sounds can significantly reduce mating rates^28^. We also checked whether the male had an effect on the calling behavior of the female. As a result, we also found that the presence of males significantly affects the calling behavior of females (Fig. 6). Therefore, we believe that NTL plays an important role in the male response to the female. On the other hand, we observed that the duration of mating and the number of eggs laid were not affected in our experiments (Fig. S2). In contrast, Wang et al*.*^19^ reported that the knocking down of NTL expression in *S. litura* adults did significantly reduce the reproductive behavior in the adults, including female calling, male courtship, mating, and also the reproductive output was dramatically affected with a reduction of more than 70% of the egg production. Such decrease in reproduction is significant and therefore may represent a potential target for pest control. Here, it should be remarked that the authors^19^ did use another method of gene-silencing; they used a bacteria-mediated RNAi approach and realized 79% of silencing of NTL, which is stronger than the 60% of RNAi efficacy in our assays with the SPc nanocarrier. As a consequence, we believe that further optimization in RNAi efficacy, and potentially also tissue-specific CRISPR-Cas9^29,30^, may help to further investigate in depth the mechanisms of NTL in sexual behavior and also egg production and oviposition.

In summary, in this study, we identified and described the molecular characteristics of NTL in the important pest insect *S. frugiperda*. In RNAi assays, dsRNA-NTL affected the reproductive behavior of *S. frugiperda* male and female adults, confirming the involvement of NTL in mating with the female calling and the male courtship rates. Our results not only provide further insights into the role of NTL in sexual behavior, which further promotes the elucidation of the physiological mechanism of NTL in insects. But, since NTL is present arthropods, including insects, the data provide also a foundation to employ NTL, regulating the sexual activity in insects, as new potential and insect-specific target to keep the populations of important pest insects as *S. frugiperda* under control based on a sterility insect technique (SIT) approach. In detail, researchers can now further investigate the potential of synthetic peptidomimetics with agonists/antagonists of NTL and novel control agents against pest *Spodoptera* together with their biosafety profile towards beneficial insects as natural enemies and pollinators^31,32^. Also future developments can look to the utilization of CRISPR/Cas9 based approaches, as the protocol to knock-out genes in Lepidoptera and *S. frugiperda* is available^30,33^, employing NTL as target in an essential insect-specific gene-directed SIT to keep these important *Spodoptera* pest populations under control.

### Supplementary Information

Supplementary Information.

## Data Availability

All data generated or analyzed during this study are included in this published article (and its Supplementary Information files).
