# Supplementary material for: Neuropeptide natalisin regulates reproductive behaviors in Spodoptera frugiperda
Source: Sci Rep. 2024 Jul 2;14:15122. doi: 10.1038/s41598-024-66031-y (PMC11220091; doi:10.1038/s41598-024-66031-y)
Supplement: Supplementary file 1 — Supplementary Information. [file 41598_2024_66031_MOESM1_ESM.docx]

**Supplementary data**


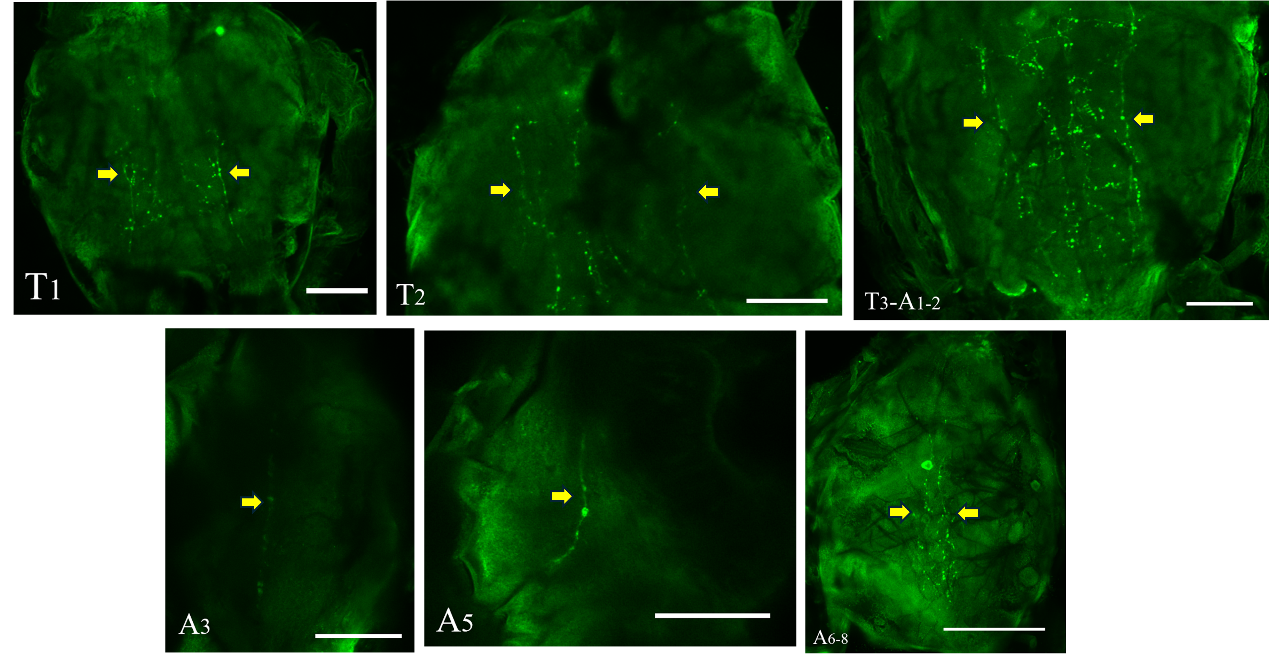


**Figure S1.** Localization of SfNTL in ventral nerve cord of *Spodoptera frugiperda*. The scale is 100 μm.


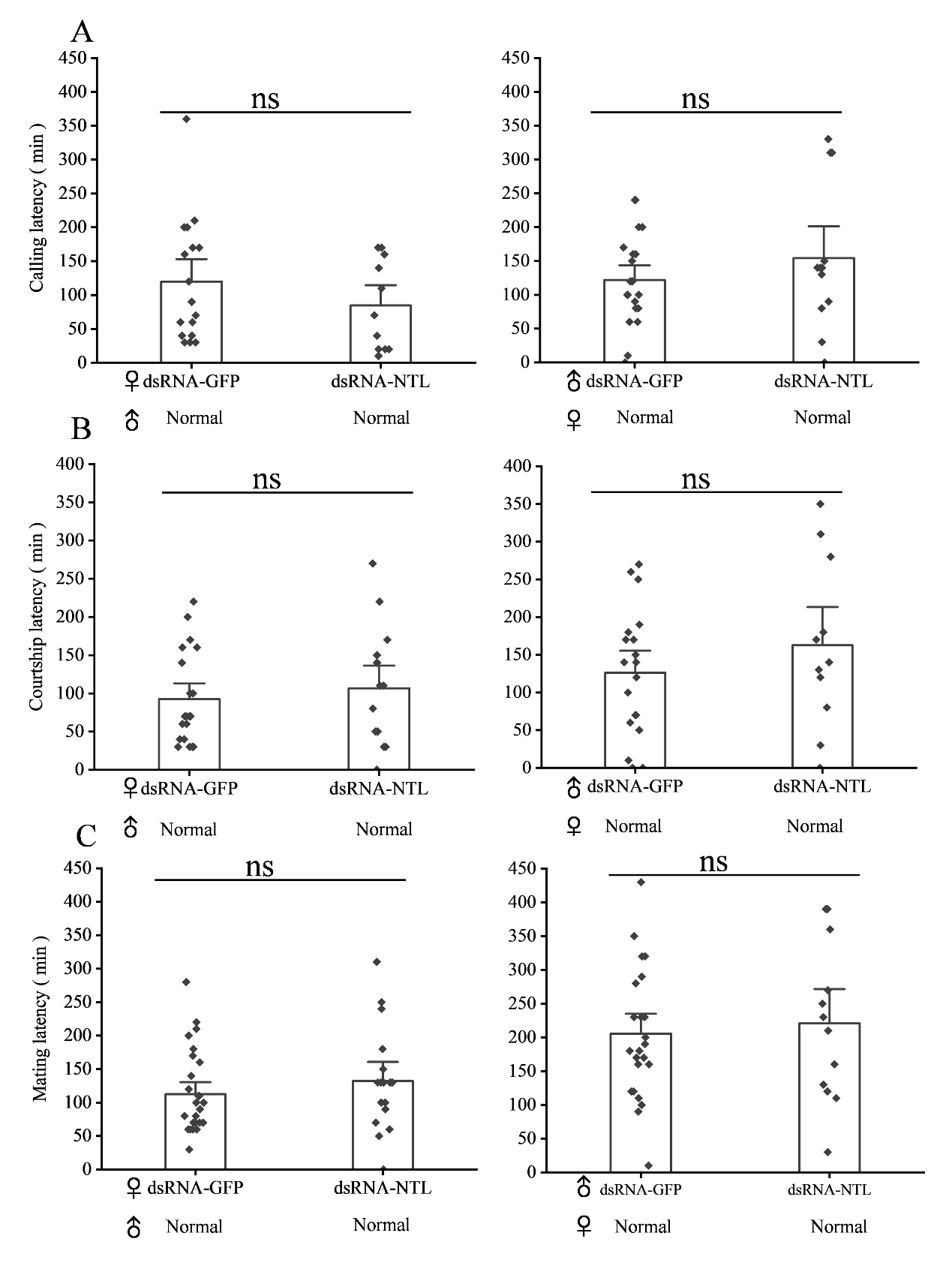
**Figure S2.** (A) The effect on the calling latency after interfering with the male or female. (B) The effect on the courtship latency after interfering with male or female. (C) The effect on mating latency after interfering with male or female. The data were analyzed by independent samples t-tests. ns, no significance


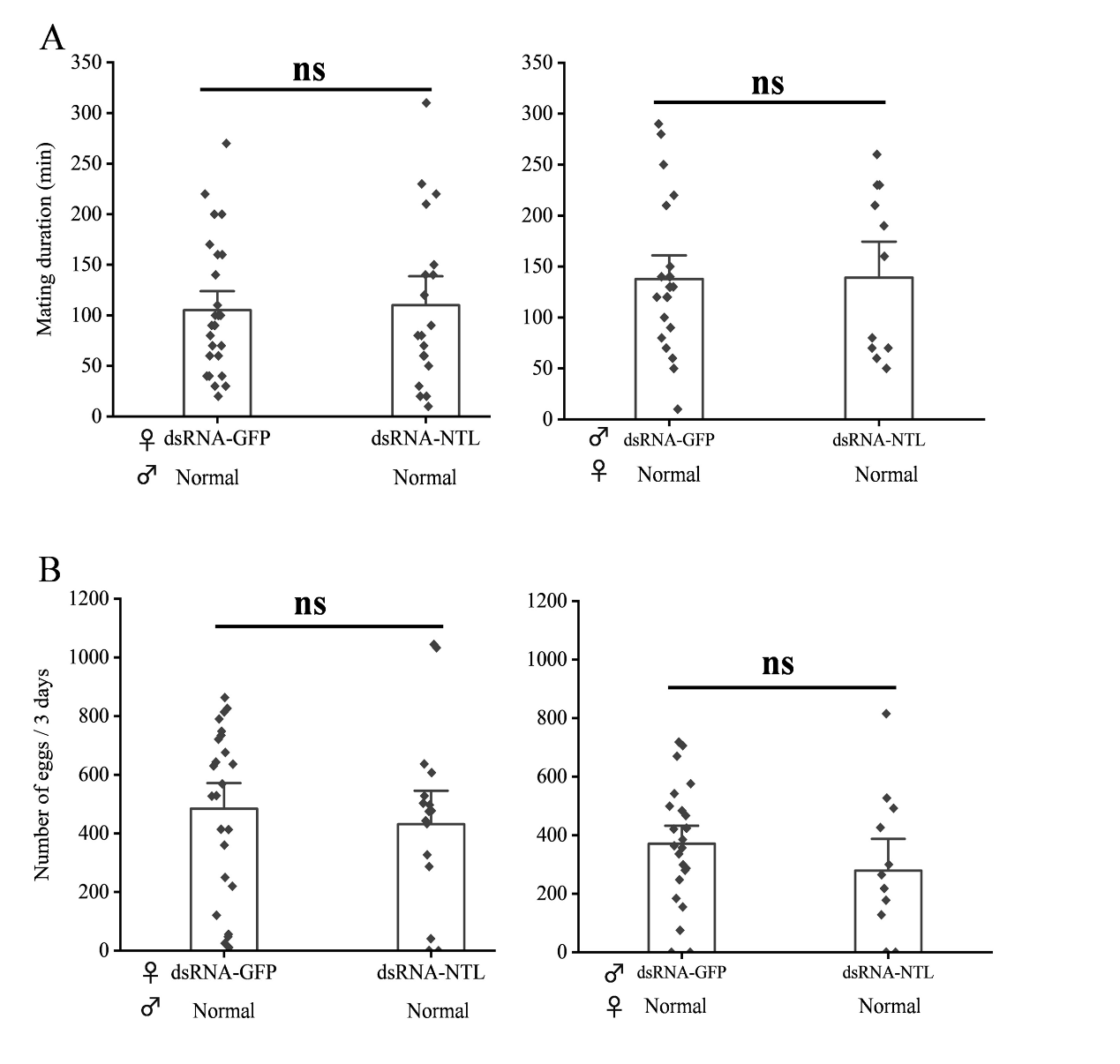


**Figure S3.** The effects of natalisin double-stranded DNA (dsRNA-NTL) injection on the mating duration and fecundity of *Spodoptera frugiperd* adults. The data were analyzed by independent samples t-tests. ns, no significance. (A) Mating duration of dsRNA-NTL adults and untreated unmated adults. (B) The number of eggs laid by a single female 3 days after mating of dsRNA-NTL adults with untreated unmated adults.

**Table S1.** Primer sequences used in this study.

|  | nucleotide sequence | |
| --- | --- | --- |
| genes | Forward | Reverse |
| qPCR-SfNTL | GGGTTCGCAAGCAAGATTTA | TTCCTGGTTCCGTAGTTTGG |
| qPCR-GAPDH | AGATCGCTGTCTTCTGCGAG | CAGACGCCTTCTCTGTGGTT |
| qPCR-RPL10 | TGGGTAAGAAGAAGGCTACG | TGTTGATGCGGATGACAT |
| dsRNA-NTL | TAATACGACTCACTATAGGGACAGAGCCAATCTACCCAAGG | TAATACGACTCACTATAGGGTTTCTTGCCCCTACTCACGA |
| dsRNA-GFP | TAATACGACTCACTATAGGGTGGGCACAAATTTTCTGTC | TAATACGACTCACTATAGGGAAGGGTATCACCTTCAAAC |

Note: The red font is the T7 promoter
